# Recurrent Large Extrauterine Lipoleiomyoma in a Postmenopausal Woman: A Case Report

**DOI:** 10.7759/cureus.43193

**Published:** 2023-08-09

**Authors:** Joshua S Braganza, Madison R Wilson, Zi L Huang, Diane Shih-Della Penna, Dennis E Johnson

**Affiliations:** 1 General Surgery, WellSpan York Hospital, York, USA; 2 Medical Education, St. George's University School of Medicine, St. George, GRD; 3 Surgery, WellSpan York Hospital, York, USA; 4 Surgical Oncology, WellSpan York Hospital, York, USA

**Keywords:** general surgery, lipoleiomyoma, gynecology oncology, pathology, surgical oncology

## Abstract

Lipoleiomyoma is a type of tumor usually found in the uterine corpus. The pathophysiology is unclear; however, it is commonly seen in obese perimenopausal and postmenopausal women. While intrauterine lipoleiomyoma may be surveilled, there is less information about the management of extrauterine lipoleiomyoma, especially significantly large tumors.

This is a case involving a 51-year-old female who was incidentally found to have a 23-cm extrauterine lipoleiomyoma emanating from the peritoneum between uterosacral ligaments. She underwent hand-assisted laparoscopic removal of an intra-abdominal tumor, which was found to be an extrauterine lipoleiomyoma. Six months later, she was found to have a recurrent mass on a follow-up computed tomography (CT) of the abdomen and pelvis. She underwent a robotic-assisted total abdominal hysterectomy, bilateral salpingo-oophorectomy, and removal of the recurrent tumor.

While the mass is benign in nature, the mass effect that it may cause prompts a discussion about the best course of management and an investigation into recurrence rates, specifically in similar extrauterine presentations.

## Introduction

Lipoleiomyoma is a rare subtype of leiomyoma composed of a significant amount of adipose and smooth muscle tissue commonly discovered in the uterine corpus [1]. The origins are unclear, primarily due to the complete absence of adipose in a healthy, functioning uterus. Despite assumptions that this is metaplasia of the leiomyoma [1], there has been research to propose monoclonality of the lipoleiomyoma [2]. Upon investigation of the tumor using an ultrasound, the lipoleiomyoma is commonly found to have a uniform echogenic profile and often without vascular content. Visual exploration will show a characteristic yellow glimmer of the tumor, where most diagnoses are made [3]. A biopsy with histological analysis is required to make an accurate diagnosis of the lipoleiomyoma. Although the most common location for this tumor is the uterine corpus [1,4], it has been found in other sites, including the breast [5], the broad ligament of the uterus [6], and intraperitoneal [7]. Most patients will present with abdominal cramping and symptoms similar to a leiomyoma, including pelvic pain, a noticeably enlarged mass of the abdomen, and dysmenorrhea. Additionally, the growth rate of the lipoleiomyoma is significantly higher than that of the leiomyoma [1,7-8]. 

A retrospective study of 76 cases showed that a significant number, 75.7%, had additional medical diagnoses due to excess estrogen within the body, including endometriosis, polyps, and endometrial hyperplasia [1]. The leiomyoma has been proven to respond to estrogen with an increased growth rate and, contrarily, regression post-menopause. Postmenopausal treatment with estrogen could be a contributing factor to the beginning and evolution of lipoleiomyoma [8]. This article was created to illuminate the data and discuss the rare yet significant development of an extrauterine lipoleiomyoma within the peritoneal space. 

## Case presentation

**Figure 1:**
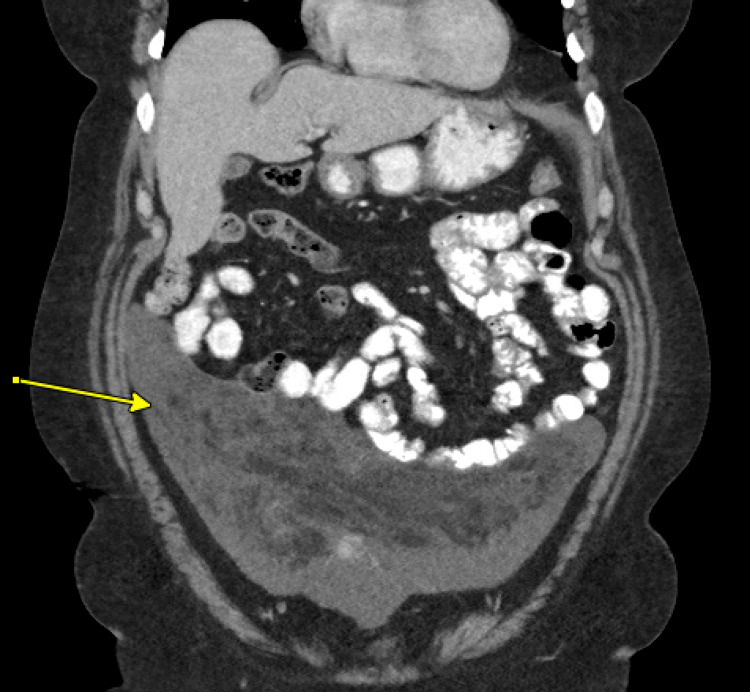
Initial CT scan demonstrating the incidentally found mass, as depicted by the arrow

A 51-year-old female was referred to our surgical oncology clinic for evaluation of a large mass in the anterior abdomen found incidentally on a CT scan in relation to a mechanical fall, as seen in Figure 1. At the time of the patient's presentation to the surgical oncology clinic, her last menstruation was approximately six months prior, and she had not been on any hormonal replacement therapy. Her BMI was 35.24 kg/m2. She had no abdominal pain, early satiety, diminished appetite, or weight loss. The patient was initially referred to interventional radiology by her primary care physician for a biopsy, which was performed using an 18-gauge tru-cut needle to obtain a pathology-confirmed diagnosis via a minimally invasive method. The results of the biopsy revealed a mesenchymal neoplasm with myxoid differentiation and a fat component that, in consultation with the pathology team from Cleveland Clinic, was benign in nature.

**Figure 2:**
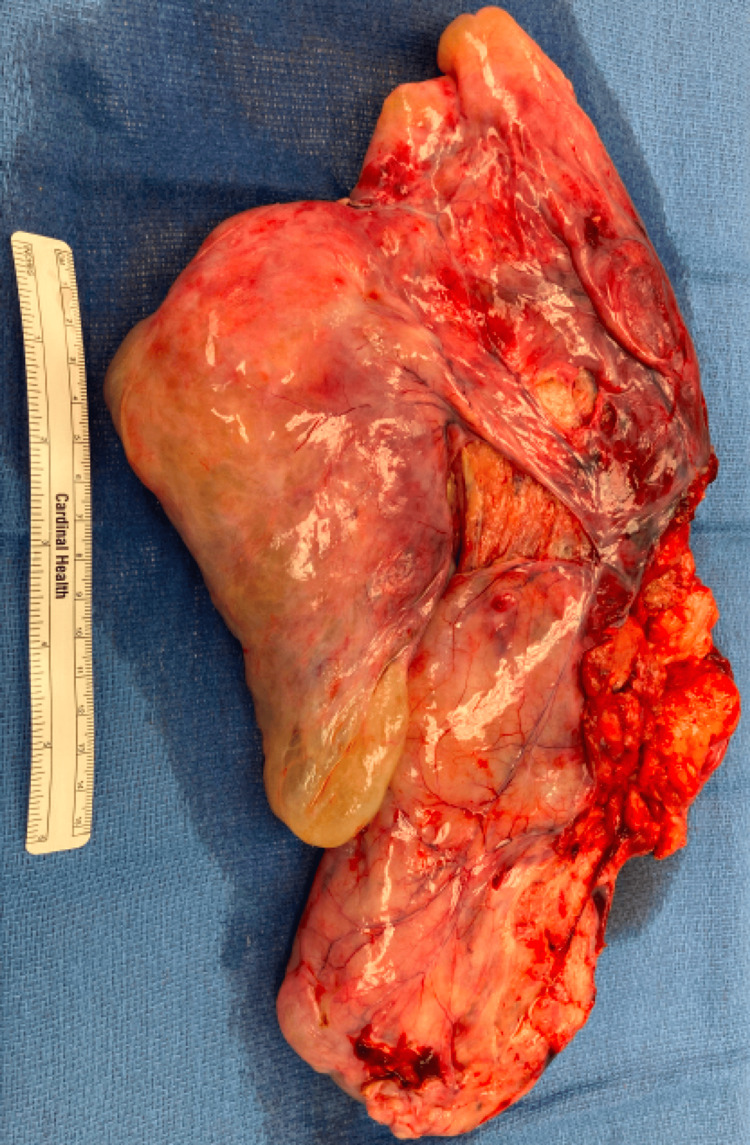
Morsel of resected mass

Given the size of the mass, with the width extending from right to left paracolic gutters, the decision was made to proceed with a diagnostic laparoscopy and subsequent resection of the mass with concern for a mass effect should the mass continue to grow. Using the Hasson technique, our initial port was placed, and insufflation was achieved. We then placed two 5 mm ports in the right mid- and lower quadrants and a 5 mm port in the left lower quadrant. Adhesions were lysed, and the mass was examined to assess the extent and origin of the mass. The omentum was retracted by the cephalad, and the origin of the mass seemed to be the peritoneum between the uterosacral ligaments spanning greater than 20 cm. An attempt was made to decompress the mass; however, upon insertion of the needle, the materials were found to be myxoid and gelatinous. Given the size of the lesion, an upper midline hand port was placed, and the mass was removed through the hand point after tedious dissection to identify the stalk between the uterosacral ligaments. The mass is pictured in Figure 2. The peritoneal defect was sutured closed laparoscopically, and the pathology was again sent to the Cleveland Clinic with the diagnosis of extrauterine lipoleiomyoma. 

On the six-month follow-up, a repeat CT scan was obtained, showing a large pelvic mass of approximately 9cm x 5cm, which was thought to be a recurrence, as seen in Figure 2. She was evaluated and booked for surgery by the gynecologic oncology team and underwent robotic assisted total abdominal hysterectomy, bilateral salpingo-oophorectomy, and resection of the mass. Again, the pathology was found to be extrauterine lipoleiomyoma. There was no evidence of endometriosis or hyperplasia; however, there was a subserosal nodule of adenomyosis. Her ovaries were noted to have benign epithelial cysts, and her fallopian tubes were noted to have para-tubal cysts.

**Figure 3:**
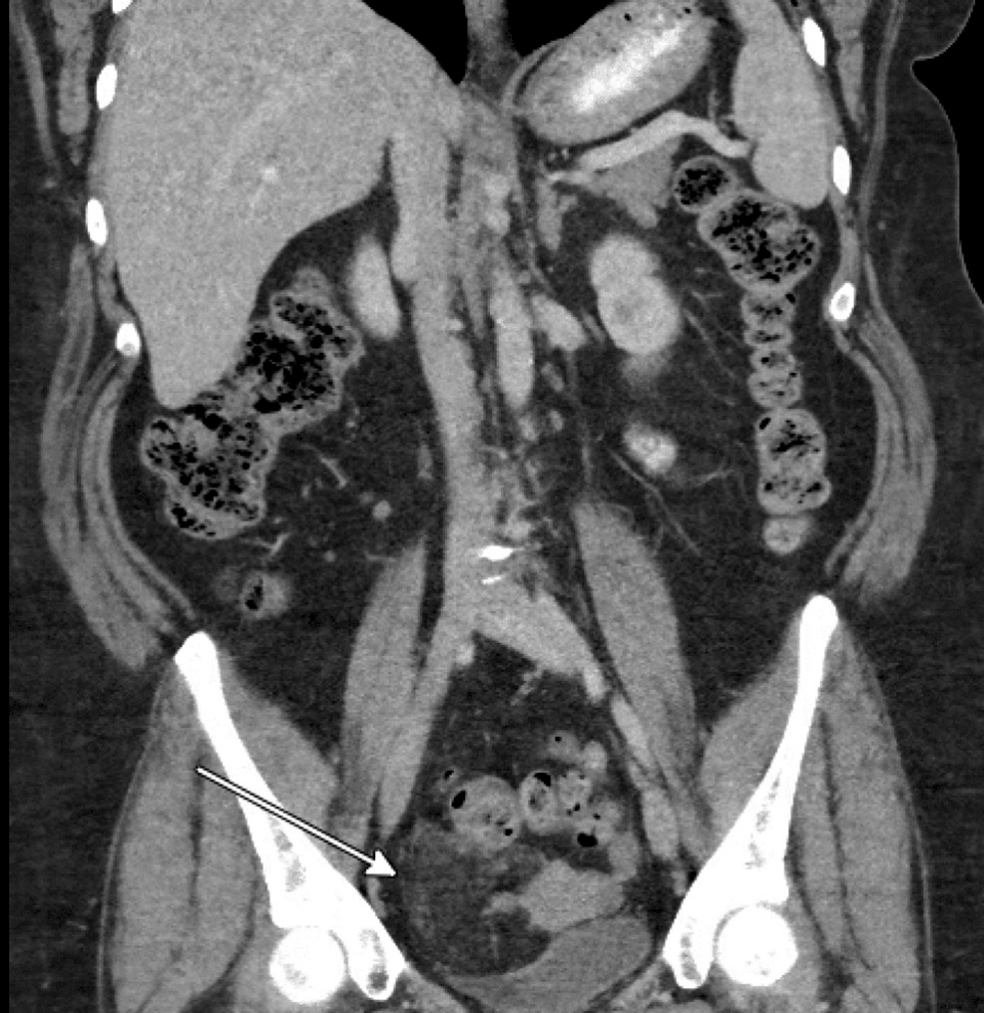
Radiographic recurrence of mass with an arrow depicting the area of concern

## Discussion

In a literature review on lipoleiomyoma, commonality across described cases was found in a uterine source as well as a perimenopausal and postmenopausal female population [9]. Interestingly, no cases have ever been described of a lipoleiomyoma emanating from the peritoneum. Differential diagnoses would include teratoma, pelvic lipoma, liposarcoma, lipoleiomyosarcoma, and ovarian tumors. 

Lipoleiomyoma has characteristic histologic findings composed of smooth muscle and mature adipose tissue [1], similar to myolipoma. The difference between the two is the location, with lipoleiomyomas mostly found in the uterus, with rare cases found in the cervix [10] and ovaries [11]. These tumors are thought to result from a fatty metaplastic process as opposed to fatty degeneration [1]. 

The pathogenesis remains unclear. Previously performed immunohistochemical studies revealed mesenchymal immature cells or the direct transformation of smooth muscle cells into adipocytes [8]. Dyslipidemic conditions associated with estrogen deficiency seen in the perimenopausal and postmenopausal periods [9] may also be a contributing factor. 

Current literature suggests that intrauterine lipoleiomyomas, when asymptomatic, require no treatment [8] and are clinically similar to leiomyomas. In this case, the mass effect alone brought up concerns that this tumor could lead to compression of intraperitoneal structures and cause a number of different pathologies, including bowel obstruction and hernia. With the recurrence of the tumor at this point, it is unclear what the appropriate management is; however, with the removal of the mass along with a hysterectomy and salpingo-oophorectomy, we hope to have a curative result for our patient. 

## Conclusions

Lipoleiomyoma is characteristically composed of smooth muscle and adipose tissue and is a very rare variant of leiomyoma. The benign tumor is most often discovered within the uterus in perimenopausal or postmenopausal women and is rarely found elsewhere. The novel presentation of this patient warrants further investigation into the pathogenesis of this disease. The current management guidelines fail to indicate the ideal surgery for symptomatic presentations, including a mass effect. Additionally, the management of recurrence has not been clearly outlined, which warrants further investigation as long-term outcomes have not been fully studied.
